# Being a radiation oncologist: times of crisis for European graduates

**DOI:** 10.1093/bjro/tzaf016

**Published:** 2025-06-09

**Authors:** Federico Gagliardi, Emma D’Ippolito, Roberta Grassi, Angelo Sangiovanni, Vittorio Salvatore Menditti, Dino Rubini, Paolo Gallo, Luca D’Ambrosio, Massimo Minerva, Viola Salvestrini, Francesca De Felice, Giuseppe Carlo Iorio, Antonio Piras, Luca Nicosia, Gian Marco Petrianni, Luca Boldrini, Valerio Nardone

**Affiliations:** Department of Precision Medicine, University of Campania “L. Vanvitelli”, Naples 80138, Italy; Department of Precision Medicine, University of Campania “L. Vanvitelli”, Naples 80138, Italy; Department of Precision Medicine, University of Campania “L. Vanvitelli”, Naples 80138, Italy; Department of Precision Medicine, University of Campania “L. Vanvitelli”, Naples 80138, Italy; Department of Precision Medicine, University of Campania “L. Vanvitelli”, Naples 80138, Italy; Department of Precision Medicine, University of Campania “L. Vanvitelli”, Naples 80138, Italy; Department of Precision Medicine, University of Campania “L. Vanvitelli”, Naples 80138, Italy; Department of Precision Medicine, University of Campania “L. Vanvitelli”, Naples 80138, Italy; School of Medicine, Università Vita-Salute San Raffaele, Milan 20132, Italy; Radiation Oncology Unit, Azienda Ospedaliero-Universitaria Careggi, University of Florence, Florence 50110, Italy; Department of Radiotherapy, Policlinico Umberto I, Sapienza University of Rome, Rome 00161, Italy; Department of Oncology, Radiation Oncology, University of Turin, Turin 10126, Italy; UO Radioterapia Oncologica, Villa Santa Teresa, Bagheria 90011, Italy; Advanced Radiation Oncology Department, IRCCS Sacro Cuore Don Calabria Hospital, Cancer Care Center, Verona 37034, Italy; Operative Research Unit of Radiation Oncology, Fondazione Policlinico Universitario Campus Bio-Medico, Via Alvaro del Portillo, 200, Roma 00128, Italy; Radiation Oncology, Fondazione Policlinico Universitario A. Gemelli, IRCCS, Largo Agostino Gemelli, Rome 00168, Italy; Department of Precision Medicine, University of Campania “L. Vanvitelli”, Naples 80138, Italy

**Keywords:** radiotherapy, residency training, Healthcare policy, European oncology, European radiotherapy, Italian radiotherapy

## Abstract

This study examines the shortage of radiation oncologists in Italy and Europe, analysing systemic challenges in postgraduate training and proposing solutions to enhance the appeal of radiation oncology. A review of literature from Italy and Europe evaluated trends in training programmes, workforce dynamics. Analysis included residency vacancies, economic constraints, training disparities, and visibility of the field during medical education. In Italy, 55.3% of radiation oncology residency positions have gone unfilled or been abandoned since 2016, with 90% of positions vacant in 2023. Contributing factors include inadequate exposure to radiotherapy during medical training, limited financial opportunities, negative societal perceptions, and high levels of burnout. Across Europe, similar challenges persist. Training disparities, outdated infrastructure, and regional inequalities exacerbate workforce shortages, particularly in low-income countries. Addressing the radiation oncology crisis requires a multifaceted strategy, including enhancing visibility of the field in medical education, improving working conditions, offering financial incentives, and addressing disparities in training quality across Europe. The European radiotherapist shortage is a systemic issue requiring coordinated efforts to standardize training, address economic barriers, and improve the specialty’s appeal. By fostering collaboration and reform, European nations can meet the growing demand for cancer care and secure a sustainable workforce for the future.

## Introduction

Cancer has a significant impact on the European population, and the rising incidence of cancer has a devastating effect not only on the families and friends of patients, but also on national healthcare systems, particularly in low-income countries.^1^

Over the past few decades, medicine has made enormous advances in the treatment of cancer, turning a once devastating diagnosis into a condition with improving survival rates. In this evolving field, the role of the radiation oncologist is crucial and has acquired a progressive importance: today radiotherapy is used in around 50% of cancer treatments worldwide, often in combination with surgery and chemotherapy.^2^

Despite the critical importance of radiotherapy and the obvious need for qualified specialists, young graduates in Europe who choose this path face significant challenges. Indeed, the radiation oncology profession is facing an unprecedented crisis due to a combination of structural, economic, and organizational factors.^3^

In the context of an ongoing crisis, a paradoxical situation has emerged. Despite an increase in the demand for radiation therapy, driven by the rising incidence of cancer and an ageing population, and the unprecedent treatment possibilities due to innovative technologies, the number of radiation oncologists in Europe is insufficient to meet this demand.

The shortage of residency positions, when considered alongside the increasingly competitive nature of the labour market, is creating significant difficulties for those new to the profession.^4^

The demand for radiation oncologists is undergoing a gradual but consistent increase, yet the training and employment opportunities available to them are not keeping pace with this growth. This imbalance could have a negative impact on the quality of oncology care that patients receive. Furthermore, the situation is further complicated by the increasing complexity of the technologies used in radiotherapy, which require continuous and specialized training.^5^

To address this crisis effectively, it is crucial to comprehend its underlying causes and to formulate efficacious strategies for its resolution.

The objective of this paper is to analyse the current challenges facing radiation oncology graduates in Europe and to explore potential solutions that could enhance their prospects. By undertaking a comprehensive analysis of the available data, we will endeavour to present a detailed overview of the current situation and potential avenues for improvement.

## Radiation oncology residents in Europe

To gain a deeper understanding of the current state of radiation oncology training in Europe a comprehensive analysis of the educational practices employed in the training of radiation oncologists across various European countries was conducted.^6^

This analysis encompassed a detailed examination of multiple aspects of radiation oncology training, including the structure of training courses, teaching methods, available resources for educational purposes, and student satisfaction.

The survey findings indicate that, despite ongoing efforts to standardize radiation oncology training, significant discrepancies remain between European countries. For instance, some countries offer training courses that are more extensive and comprehensive, while others provide shorter and less rigorous ones. These discrepancies have the potential to affect the quality of training, which, in turn, may influence the standard of care provided to patients. Furthermore, the report highlighted the need to expand training capacity and improve access to educational resources, with the goal of ensuring that all radiation oncology graduates receive adequate and comprehensive training. Student satisfaction levels also vary considerably, with some students reporting a lack of sufficient resources and internship opportunities.

The results of European studies indicate that there is a complex relationship between satisfaction with one’s career choice, concern about the quality of training, and perceptions of career prospects.^7–9^

A key issue that emerges from all 3 sources is the lack of visibility of radiation therapy during medical school. This lack of familiarity with radiation therapy results in a restricted perception of its potential and the career opportunities it offers.

A further crucial aspect is the quality and duration of the training received. Indeed, there is a significant discrepancy between satisfaction with the chosen specialty and satisfaction with the training received.

Furthermore, the absence of integration between clinical and research activities was identified as a limitation to career prospects, particularly for those pursuing an academic trajectory.

The issue is not so much the content of radiation oncology specialization courses, but rather the consistent decline in the number of new graduates opting for this specialty. Each year, an increasing number of physicians opt to pursue opportunities in other medical specialties, resulting in a growing number of vacant radiation oncology residency positions. This trend has the potential to exacerbate the shortage of radiation oncologists, despite the increase in the number of fellowships and training opportunities available.

The current crisis has had significant repercussions in many European countries, as evidenced by the experience of Italy, where the scholarship number among all the different specialties was indiscriminately increased without accounting for the real requirement of National Healthcare System. Consequently, despite this growth, a considerable number of applicants have selected alternative specializations in national competitions, resulting in radiation oncology becoming one of the least competitive disciplines in terms of enrolment.

Concurrently, there has been a notable decline in the number of fellowships available in radiation oncology specialization schools, which can be attributed to 2 primary factors. Firstly, some fellowships remain unallocated due to their non-selection by the successful candidates in the subsequent stages of the selection process. Secondly, a proportion of medical residents have opted to transition out of the radiation oncology programme, pursuing opportunities in other specialties during their training.

Over the past 3 years, a significant number of radiation oncology fellowships have remained unoccupied or have been relinquished. This gradual decline in the ratio of applicants to available fellowships has resulted in an increase in the number of unallocated or abandoned fellowships.^10^

Similarly, Spain is examining the potential implications of staffing shortages and the capacity to meet the increasing demand for radiation therapies. As the number of cancer cases is expected to increase, the need for radiotherapy services is growing. However, a shortage of radiation oncologists and outdated equipment may impede the ability to provide high-quality treatment.

Despite the opening of new radiotherapy centres and the expansion of services at some hospitals, these efforts are insufficient to meet the growing demand. The workforce gap is projected to widen as the number of oncologists required to treat the growing number of patients and accommodate advances in treatment techniques increases. Furthermore, discrepancies in the availability of staff and equipment across regions intensify the situation, with rural areas facing the most significant challenges.^11^

Additionally, Poland presents a comprehensive analysis of radiation oncology training, elucidating both the strengths and challenges encountered by trainees. While most respondents expressed satisfaction with their training hospitals, a considerable proportion reported feelings of being overworked.

The training course itself, which includes compulsory courses and internships, has been the subject of criticism on the grounds that its content is repetitive and that it lacks sufficient focus on modern advances.^7^

## Radiation oncology residents in Italy

Since 2014, all Italian Residency Schools have participated in a single national competition. Each year, the Ministry of University and Research publishes a single announcement, establishing the number of available positions for each specialty and distributing them among the various Residency Schools across the country.

**Figure 1.**
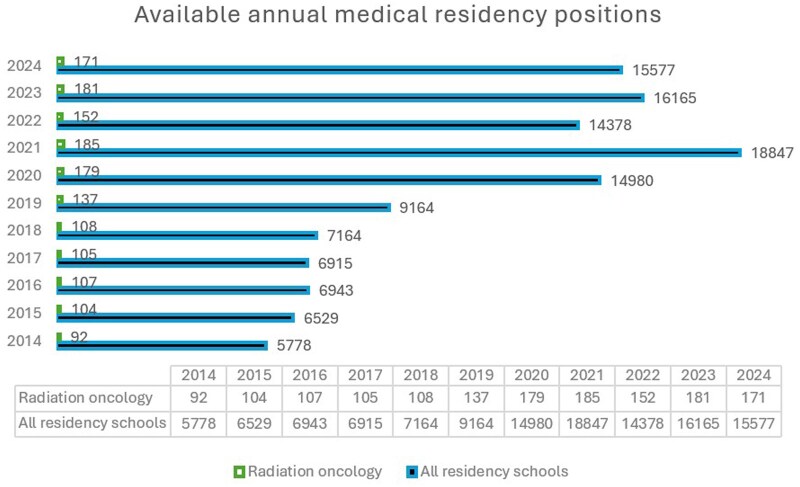
Available annual medical residency positions.

According to data from the Ministry, postgraduate medical training in Italy has undergone significant changes over the past 10 years. Since 2020, in response to the post-COVID-19 healthcare crisis, the number of available residency positions has increased significantly, impacting admissions across all specialties, including the radiation oncology residency schools (Figure 1).

**Figure 2.**
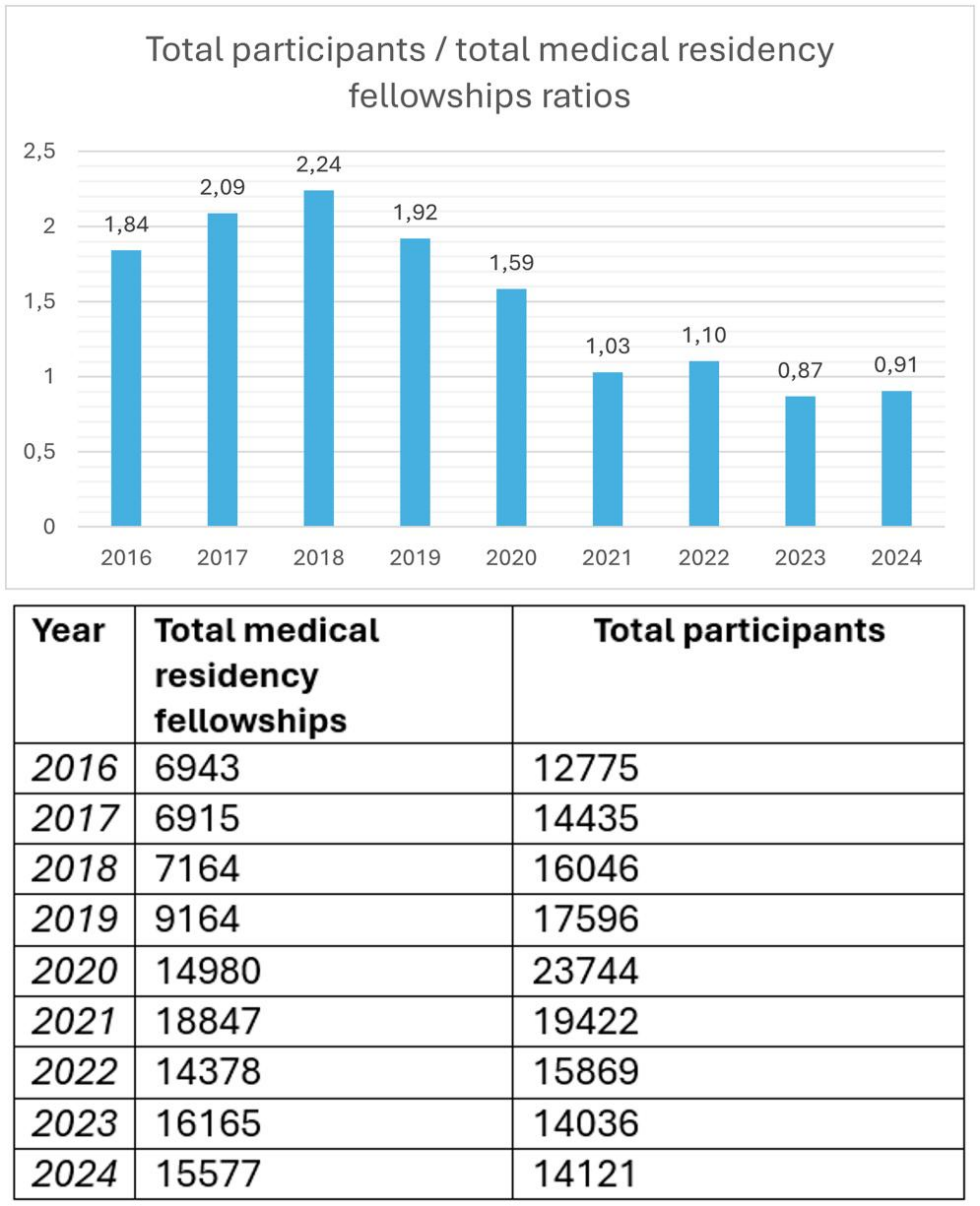
Number of total participants and available medical residency positions in Italy from 2016 to 2024, along with their ratio.

As a result, the number of available medical residency scholarships exceeded the number of applicants. Figure 2 illustrates how the ratio between available medical residency scholarships and the number of applicants has evolved over time.

Focusing on the last 2 years, there were 14 036 participants for 16 165 residency positions in 2023 and 14 121 participants for 15 577 residency positions in 2024.

This imbalance has resulted in a situation where fewer medical residents are entering into less popular specialties, such as radiation oncology, nuclear medicine, medical pathology, microbiology, anatomical pathology, and pharmacology. Conversely, highly desired specialties, including dermatology and cardiology, have received a significantly higher number of applications than there are available positions. Specialties such as radiation oncology and nuclear medicine are often perceived as offering limited career opportunities in the private sector, as they typically require a long-term commitment to the public healthcare system. This may act as a deterrent to those seeking greater career flexibility.

In Italy, radiation oncology residency programmes have historically faced both a high rate of unfilled positions and a significant dropout rate, as many residents leave to pursue more competitive specialties. In 2016, 11 out of 107 positions (10.3%) were unfilled or abandoned; in 2017, 20 out of 105 (19%); in 2018, 31 out of 108 (28.7%); and in 2019, 42 out of 137 (30.7%).^5^

Over the years, the distribution of radiation oncology residency positions has changed, partly due to shifts in the ranking list and doctors reapplying to change specialties, as seen in the studies by Nardone et al^5^ and Petrianni et al.^4^

In 2020, the Nardone et al study reported 50 lost radiation oncology residency positions, but the actual number was 72 out of 179 (40.2%), with 22 trainees dropping out of the programmes. In 2021, the number of lost positions increased from the 96 reported in Nardone et al to 119 out of 185 (64.3%). The number of abandoned positions rose from 6 to 30, while unassigned positions decreased from 90 to 89, compared to the previous study.

In 2022, the distribution shifted again, with 124 out of 152 (81.6%) positions lost, of which 111 (73%) remained unassigned, 13 (8.6%) were abandoned, and only 28 (18.4%) were assigned.

In 2023, the number of lost positions reached 163 out of 181 (90%), with 158 (87.3%) unassigned, 5 (2.7%) abandoned, and only 18 (10%) assigned.

The latest competition for residency placements, which concluded in November 2024, saw 171 available positions, with 151 (88.3%) unassigned and only 20 (11.7%) assigned.

Since 2016, a total of 733 out of 1,325 (55.3%) radiation oncology residency positions have been lost.^12^

**Figure 3.**
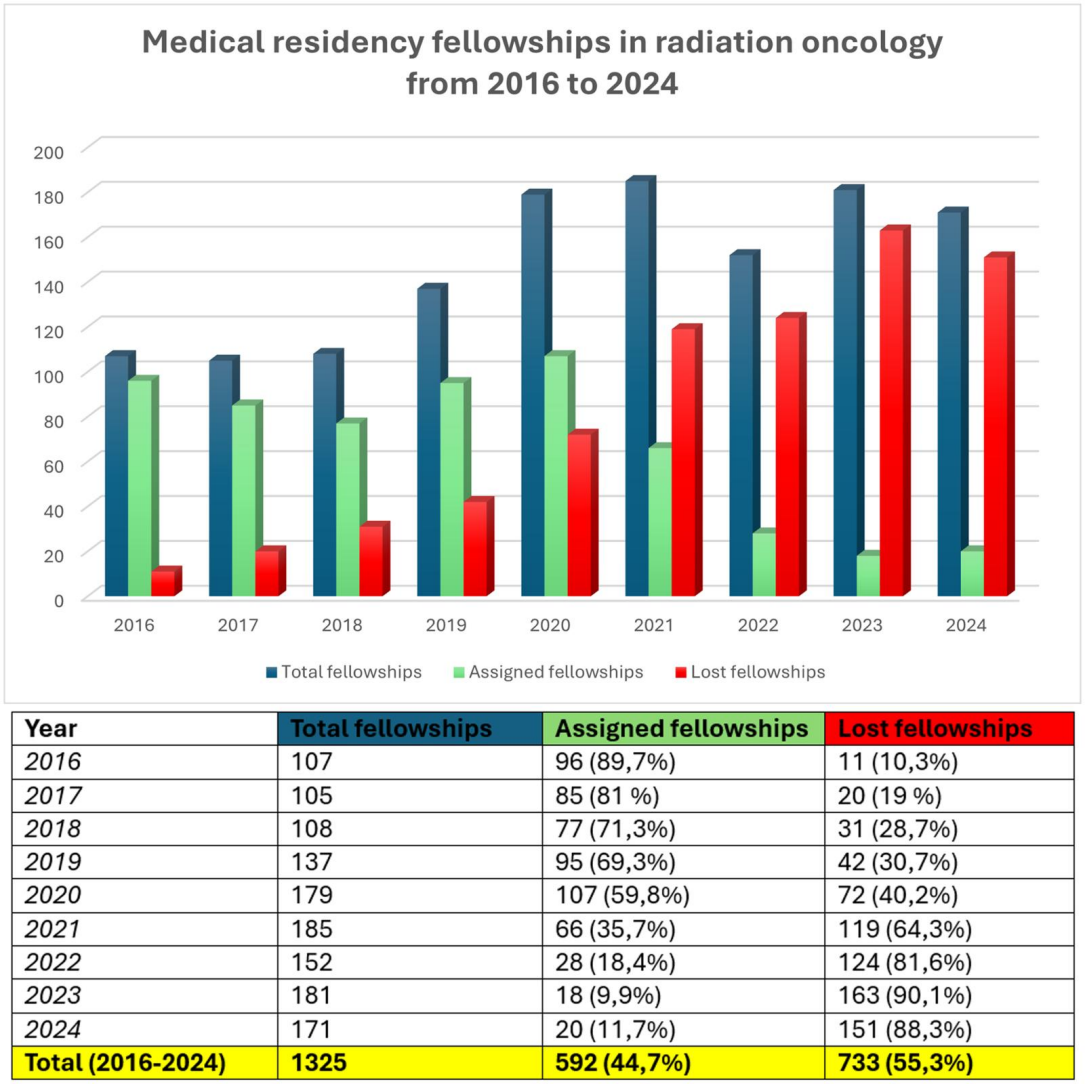
Summary of residency positions in radiation oncology from 2016 to 2024, showing the percentage of assigned and unassigned positions over the years.

Figure 3 summarizes the number of assigned, unassigned, and abandoned positions over the past 9 years in Italy.

## The wrong question

It is imperative to consider whether the current approach to addressing the shortage of radiation oncology graduates is indeed the most effective. Rather than focusing on the structure or content of training programmes, it may be more beneficial to investigate the underlying reasons behind the growing reluctance of medical graduates to pursue radiotherapy as their initial career choice. By identifying and addressing the root causes, which could be related to working conditions, job satisfaction or career prospects, we may be able to develop strategies to reverse this trend and attract more talent to the field.

There is now a widespread recognition that the field of radiation oncology is facing an imminent crisis due to an increasing deficit in specialists. Nevertheless, the challenge extends beyond mere recognition; it also encompasses an understanding of how to enhance the appeal of the specialty to new graduates.^4^

In Italy, the deficit of radiation oncology trainees may be attributed to economic factors, as the field offers limited prospects for private practice, rendering it less financially lucrative than other medical specialties. Furthermore, the absence of dedicated radiation oncology courses at the university level contributes to this issue, as the lack of early exposure to the field results in many medical students receiving an incomplete introduction to the discipline, leading to diminished interest when it comes time to select a specialization (https://www.corriere.it/salute/24_marzo_10/radioterapia-in-carenza-di-medici-be5f04ff-ce43-458b-9a01-b5ea7b59axlk.shtml?refresh_ce.2024).

It is conceivable that in the past, when the number of postgraduate fellowships was lower than the number of new graduates available, all the places were filled, resulting in some individuals being compelled to pursue a career in radiation oncology. Many of these individuals, upon commencing their careers, discovered an interest and subsequently gravitated towards this discipline. In the current context, with an equal or greater number of grants for new graduates, those who do not gain admission to their preferred specialty tend to defer their application for a year, rather than pursue a specialization such as radiation oncology, with which they are unfamiliar.^5^

## The good answer

The global shortage of radiation oncologists is a growing concern that has emerged in various regions worldwide, including Europe.^13–15^

At the outset of the 2000s, projections appeared optimistic, suggesting that the deficit would be addressed within a few years due to anticipated growth in hospital infrastructure and an increase in available radiation therapies.^16^

However, the landscape has undergone a significant transformation. As the global population has expanded, the proportion of the population in older age groups has increased, and the number of new cancer diagnoses has risen, there has been a corresponding exponential growth in demand for cancer treatments, exerting considerable pressure on healthcare systems worldwide.^17^

In Canada, one of the countries facing this problem, a forecasting model was employed to predict that from 2026 onwards, the number of available radiation oncologists is expected to decline significantly, despite an anticipated increase in demand for treatment.

Without substantial intervention, the Canadian healthcare system may experience a shortage of specialists, which could negatively impact the quality of cancer care provided to patients.^18^

In Europe, despite the difficulties, efforts are being made to tackle the problem proactively. The Young Italian Association of Radiotherapy and Clinical Oncology (yAIRO), following the path set out by ESTRO Vision 2030, has taken a step in the right direction by promoting initiatives to attract medical students to the discipline.^19^^,^^20^ It has been recognized that one of the factors contributing to the shortage of specialists is the lack of visibility and attractiveness of radiation oncology among new medical graduates. Many students do not have the opportunity to adequately study or learn about the specialty during their training, which often takes a back seat to other more well-known medical disciplines.

To counter this trend, efforts are being made to promote radiation oncology in universities through workshops, seminars, and work placements that allow students to have direct contact with clinical practice. Efforts are also being made to make information about career prospects in the field more accessible and understandable, demonstrating that radiation oncology is a constantly evolving discipline that offers opportunities for professional growth and deep personal satisfaction.

Another aspect that will be improved is communication between the various European radiation oncology, oncology, and surgery associations. Their collaboration is essential to promote the exchange of knowledge and best practice, and to create a more inclusive and stimulating environment for medical students interested in radiation oncology or all oncology disciplines.^21^

Nevertheless, the mere promotion of specialization in universities is insufficient; it is also imperative to enhance the working conditions of radiation oncologists. This necessitates an augmentation of technical and administrative support within hospital departments, accompanied by a more optimal equilibrium between professional and personal lives. Radiation oncologists are confronted with considerable workloads, which engender elevated stress levels and an increased risk of burnout. These factors may prove deterrent to the pursuit of this career by young medical professionals. The implementation of improved working conditions and more transparent pathways for career advancement could render radiation oncology a more appealing option.^22^

Another factor to consider is the opinion towards radiation oncology in the general population. A recent paper analysing articles published in The New York Times between 1851 and the present day found that over half of them were critical of radiation oncology. In particular, the media shifted from describing the scientific background or results and the implementation of new techniques to reporting toxicity, ineffectiveness, and errors.^23^ A negative portrayal might discourage the choice radiation oncology from medical students that can be seen from outside as an “ineffective” and “toxic” treatment. On the contrary, in this historical moment radiotherapy is showing excellent results incomparable to the past in terms of cure rate and toxicity. Therefore, the improvement of the quality of work should go along with a change in the narrative of this discipline.

## Future perspective: enhancing the appeal of radiation oncology in Italy

To address the ongoing crisis in radiation oncology in Italy and attract more young physicians to this specialty, a multifaceted approach is essential. Below are proposed strategies to boost interest and retention in the field of radiation oncology:

1. Increase awareness of radiation oncology in medical education
  - Incorporate radiation oncology more prominently within the medical school curriculum, including dedicated lectures and modules that explain the role, impact, and innovations in radiotherapy.
  - Provide students with hands-on experience through clinical rotations and internships in radiation oncology departments, fostering early interest and awareness of the specialty.
2. Enhance visibility of radiation oncology across all audience levels
  - Initiate targeted information campaigns to educate the general public, policymakers, healthcare providers, and medical students on the benefits and advancements in radiation oncology.
  - Collaborate with media outlets to counter negative portrayals and emphasize recent breakthroughs, such as precision radiotherapy and reduced treatment toxicity, presenting radiation oncology as a safe, effective, and essential part of cancer care.
  - Develop educational materials tailored for patients, highlighting how radiotherapy complements other treatments to improve survival and quality of life.
3. Improve work conditions for radiation oncologists
  - Establish policies to ensure a better work-life balance for radiation oncologists, addressing issues such as long working hours, administrative burdens, and high patient loads.
  - Introduce measures to support flexible working hours and family-friendly policies, which can reduce burnout and increase job satisfaction.
  - Regularly review and adjust salary levels to remain competitive with other medical specialties, both to recognize the complex skill set and commitment required in radiation oncology and to compensate for the lower opportunity for private practice earnings in comparison to other fields.
4. Strengthen professional development and career growth opportunities
  - Offer robust mentorship programmes and continued professional development (CPD) opportunities to allow young radiation oncologists to specialize further, engage in research, or assume leadership roles within the field.
  - Facilitate networking and collaborative projects with international radiation oncology bodies to expand career perspectives and foster a sense of belonging within the global oncology community.
5. Engage policymakers to support and invest in radiation oncology
  - Work with healthcare policymakers to secure funding for modernizing radiotherapy equipment and increasing residency positions, making the field more attractive to graduates.
  - Advocate for government-sponsored scholarships and grants specifically for radiation oncology residents, helping alleviate financial burdens and incentivizing specialization in the field.
  - Present data-driven arguments to national and regional health authorities to underscore the importance of radiation oncology in addressing Italy’s rising cancer rates and achieving equitable cancer care.

Through these initiatives, Italy can work towards revitalizing interest in radiation oncology and ensuring a steady supply of specialists to meet the growing demand for cancer treatments.

## Discussion

The global shortage of radiation oncologists reveals common trends across countries, as well as notable differences in the approaches of professional associations. These associations are striving to enhance the appeal of radiation oncology as a specialization by implementing educational initiatives. This is intended to address the shortage through increasing awareness and visibility of the field, which has been affected by its limited visibility and the lack of opportunities for freelancing, which have made it a less popular choice among young graduates.

However, in other parts of the world, the situation is distinct. In the United States, the observed trend appears to be in opposition to this hypothesis. A review of the literature suggests that there will be a surplus of radiation oncologists in the near future, with a greater number of specialists than there will be demand for.^24^^,^^25^

This dynamic may be attributed to the existence of a private healthcare system, which may provide radiation oncologists with enhanced economic and professional opportunities. The availability of significant funding may render this career more attractive than in other countries where the public health system is the dominant force and opportunities for freelancing are limited.

It is also necessary to consider the demographic and social factors involved. While in Europe and Asia, the ageing of the population is a more evident phenomenon, in the United States the demographic distribution could lead to a different demand for oncology services, with a lower demand for radiotherapy. This would contribute to the surplus of specialists.

Furthermore, it is important to consider that in the United States, there is a combination of public and private insurance, yet a significant proportion of the population remains uninsured or underinsured. This could potentially result in a reduction in the demand for radiation treatments, as they may not be covered by health insurance, even if medically necessary.^26^

To enhance the appeal of a career in radiation oncology to prospective students, it is vital to enhance the visibility of the discipline during the training period. At present, the field of radiation oncology does not receive the same level of attention as other specializations within the curriculum of university medical programmes. The opportunity to participate in real-life treatments during internships could serve to enhance familiarity and interest in this specialization. It is hypothesized that students would be more inclined to consider a career in radiation oncology if they were to gain first-hand experience of the daily practices of a radiation oncologist, and thus gain an understanding of the complexity and importance of the treatment.

From an economic standpoint, enhancing remuneration and professional advancement prospects can have a significant impact. In comparison to other medical specializations, radiation oncology offers fewer freelance opportunities, which could be perceived as a disadvantage by those who have recently graduated. It would be prudent to ensure competitive salaries and long-term growth prospects in the public sector, as this could enhance the appeal of the specialization, particularly for those seeking economic stability.

It is essential to improve the balance between work and personal life for young physicians, as many of them place great emphasis on quality of life. A profession that allows them to balance work and free time will therefore become more attractive. The provision of working conditions that reduce stress and facilitate a more sustainable approach to workload management would serve as an additional incentive for those considering radiation oncology as a long-term career.

## Conclusion

It is evident that the deficit of radiation oncologists signifies a crisis that necessitates a unified response. Professional associations and health institutions must collaborate to enhance the appeal of this specialization, through the optimization of training, the provision of more appealing economic prospects and the promotion of the discipline from the earliest stages of the medical pathway. Only through a shared commitment will it be feasible to resolve this emergency and ensure an adequate number of specialists to meet the growing demand for cancer treatments.

## References

[1] Chen S , Cao Z, Prettner K, et al Estimates and projections of the global economic cost of 29 cancers in 204 countries and territories from 2020 to 2050. JAMA Oncol. 2023;9:465-472. 36821107 10.1001/jamaoncol.2022.7826 PMC9951101

[2] Borras JM , Barton M, Grau C, et al The impact of cancer incidence and stage on optimal utilization of radiotherapy: methodology of a population based analysis by the ESTRO-HERO project. Radiother Oncol. 2015;116:45-50. 26002304 10.1016/j.radonc.2015.04.021

[3] Lara PC , Benstead K, Erikssen JG. Training in radiation and clinical oncology in Europe. J Med Educ Curric Dev. 2023;10:23821205231197982. 37692557 10.1177/23821205231197982 PMC10483962

[4] Petrianni GM , Fiore M, Onorati E, et al Addressing the crisis: the looming shortage of radiation oncologists and the urgency for action. Radiol Med. 2023;128:1284-1285. 37368227 10.1007/s11547-023-01667-w

[5] Nardone V , Boldrini L, Salvestrini V, et al Are you planning to be a radiation oncologist? A survey by the young group of the Italian Association of Radiotherapy and Clinical Oncology (yAIRO). Radiol Med. 2022;128:252-260. 36586084 10.1007/s11547-022-01586-2

[6] Bibault J-E , Franco P, Borst GR, et al Learning radiation oncology in Europe: results of the ESTRO multidisciplinary survey. Clin Transl Radiat Oncol. 2018;9:61-67. 29594252 10.1016/j.ctro.2018.02.001 PMC5862689

[7] Napieralska A , Tomasik B, Spałek M, et al Radiation oncology training in Poland: multi-institutional survey. J Cancer Educ. 2021;36:769-778. 32052261 10.1007/s13187-020-01702-8 PMC8328852

[8] Gomis Sellés E , Montero A, Arenas M. Making radiation oncology specialty more attractive to young medical graduates: pulling back the invisibility curtain. Clin Transl Oncol. 2023;25:3312-3318. 37378794 10.1007/s12094-023-03257-8

[9] Krug D , Baumann R, Rieckmann T, et al Situation of young radiation oncologists, medical physicists and radiation biologists in German-speaking countries: results from a web-based survey of the Young DEGRO working group. Strahlenther Onkol. 2016;192:507-515. 27343188 10.1007/s00066-016-1003-y

[10] Specializzazioni: immatricolati solo 10mila giovani medici su 16 mila. Fuga da Emergenza-Urgenza e Radioterapia. Als e Anaao Giovani: subito tavolo di riforma interministeriale della formazione medica. Sanità. 2023;24. Accessed November 8, 2024. https://www.sanita24.ilsole24ore.com/art/lavoro-e-professione/2023-10-19/specializzazioni-mediche-immatricolati-solo-10mila-giovani-medici-16-mila-fuga-emergenza-urgenza-e-radioterapia-als-e-anaao-giovanisubito-tavolo-riforma-interministeriale-formazione-medica-172009.php? uuid=AFmNK9IB

[11] Rodríguez A , Arenas M, Lara PC, et al Spanish Society of Oncology and Radiotherapy (SEOR) Analysis Group. Are there enough radiation oncologists to lead the new Spanish radiotherapy? Clin Transl Oncol. 2019;21:1663-1672. 30941701 10.1007/s12094-019-02095-x

[12] Associazione Liberi Specializzandi (ALS) Fattore 2A. 2024. Accessed November 23, 2024. https://als-fattore2a.org/

[13] Wang MH , Loewen SK, Giuliani M, et al Clinical learning, didactic education, and research experiences of radiation oncology resident physicians in Canada. J Cancer Educ. 2022;37:155-162. 32621072 10.1007/s13187-020-01799-x

[14] Murakami Y , Noda S-e, Hatayama Y, et al What motivated medical students and residents to become radiation oncologists in Japan?-Questionnaire report by the radiotherapy promotion committee of JASTRO. J Radiat Res. 2020;61:727-732. 32696970 10.1093/jrr/rraa051 PMC7482163

[15] Mukherjee A , Manir KS, Basu P, et al Choosing a career in radiation oncology in India: a survey among young radiation oncologists. J Cancer Res Ther. 2021;17:231-234. 33723160 10.4103/jcrt.JCRT_779_19

[16] Morgan G , Wigg D, Childs J. Projected requirements for radiation oncologists and trainees in Australia and New Zealand to 2007. Australas Radiol. 2000;44:88-97. 10761265

[17] Smith BD , Smith GL, Hurria A, et al Future of cancer incidence in the United States: burdens upon an aging, changing nation. J Clin Oncol. 2009;27:2758-2765. 19403886 10.1200/JCO.2008.20.8983

[18] Loewen SK , Ruan Y, Wu CHD, et al Supply and demand for radiation oncologists in Canada: workforce planning projections from 2020 to 2040. Int J Radiat Oncol Biol Phys. 2024;119:756-770. 37562734 10.1016/j.ijrobp.2023.07.026

[19] De Felice F , Boldrini L, Greco C, et al ESTRO vision 2030: the young Italian Association of Radiotherapy and Clinical Oncology (yAIRO) commitment statement. Radiol Med. 2021;126:1374-1376. 34283336 10.1007/s11547-021-01398-w PMC8520506

[20] Lievens Y , Ricardi U, Poortmans P, et al Radiation oncology. Optimal health for all, together. ESTRO vision, 2030. Radiother Oncol. 2019;136:86-97. 31015134 10.1016/j.radonc.2019.03.031

[21] Pavlidis N , Madry R, Peeters M, et al in addition to a List of Contributors. ESO-ESSO-ESTRO multidisciplinary course in oncology for medical students: 4 years of experience (2016-2019). J Cancer Educ. 2022;37:1239-1244. 33387267 10.1007/s13187-020-01947-3

[22] Ciammella P , De Bari B, Fiorentino A, et al AIRO Giovani (Italian Association of Radiation Oncology-Young Members Working Group). The “BUONGIORNO” project: burnout syndrome among young Italian radiation oncologists. Cancer Invest. 2013;31:522-528. 24010828 10.3109/07357907.2013.830735

[23] Wawrzuta D , Klejdysz J, Chojnacka M. The rise of negative portrayals of radiation oncology: a textual analysis of media news. Radiother Oncol. 2024;190:110008. 37972739 10.1016/j.radonc.2023.110008

[24] Shah C , Mohindra P, Arnone A, et al The American Society for Radiation Oncology Workforce Taskforce Review of the United States Radiation Oncology Workforce Analysis. Int J Radiat Oncol Biol Phys. 2023;116:484-490. 36898417 10.1016/j.ijrobp.2023.02.056

[25] Pan HY , Haffty BG, Falit BP, et al Supply and demand for radiation oncology in the United States: updated projections for 2015 to 2025. Int J Radiat Oncol Biol Phys. 2016;96:493-500. 27209499 10.1016/j.ijrobp.2016.02.064

[26] Tulchinsky TH , Varavikova EA. Chapter 13 - National Health Systems. In: Tulchinsky TH, Varavikova EA, eds. The New Public Health. 3rd ed. Academic Press; 2014:643-728.

